# Control of the neurovascular coupling by nitric oxide-dependent regulation of astrocytic Ca^2+^ signaling

**DOI:** 10.3389/fncel.2015.00059

**Published:** 2015-03-10

**Authors:** Manuel F. Muñoz, Mariela Puebla, Xavier F. Figueroa

**Affiliations:** Facultad de Ciencias Biológicas, Departamento de Fisiología, Pontificia Universidad Católica de ChileSantiago, Chile

**Keywords:** cerebral arterioles, cerebral blood flow, connexins, pannexins, gap junctions, endothelial nitric oxide synthase, neuronal nitric oxide synthase, TRPV4 channels

## Abstract

Neuronal activity must be tightly coordinated with blood flow to keep proper brain function, which is achieved by a mechanism known as neurovascular coupling. Then, an increase in synaptic activity leads to a dilation of local parenchymal arterioles that matches the enhanced metabolic demand. Neurovascular coupling is orchestrated by astrocytes. These glial cells are located between neurons and the microvasculature, with the astrocytic endfeet ensheathing the vessels, which allows fine intercellular communication. The neurotransmitters released during neuronal activity reach astrocytic receptors and trigger a Ca^2+^ signaling that propagates to the endfeet, activating the release of vasoactive factors and arteriolar dilation. The astrocyte Ca^2+^ signaling is coordinated by gap junction channels and hemichannels formed by connexins (Cx43 and Cx30) and channels formed by pannexins (Panx-1). The neuronal activity-initiated Ca^2+^ waves are propagated among neighboring astrocytes directly via gap junctions or through ATP release via connexin hemichannels or pannexin channels. In addition, Ca^2+^ entry via connexin hemichannels or pannexin channels may participate in the regulation of the astrocyte signaling-mediated neurovascular coupling. Interestingly, nitric oxide (NO) can activate connexin hemichannel by S-nitrosylation and the Ca^2+^-dependent NO-synthesizing enzymes endothelial NO synthase (eNOS) and neuronal NOS (nNOS) are expressed in astrocytes. Therefore, the astrocytic Ca^2+^ signaling triggered in neurovascular coupling may activate NO production, which, in turn, may lead to Ca^2+^ influx through hemichannel activation. Furthermore, NO release from the hemichannels located at astrocytic endfeet may contribute to the vasodilation of parenchymal arterioles. In this review, we discuss the mechanisms involved in the regulation of the astrocytic Ca^2+^ signaling that mediates neurovascular coupling, with a special emphasis in the possible participation of NO in this process.

## Introduction

Information processing by the neuronal network in the central nervous system (CNS) is a very complex task that relies on dynamic interactions between neurons and glial cells, but also on functional association among brain cells and cerebral microcirculation, which is intended to be reflected by the concept “neurovascular unit” (Koehler et al., 2006; Abbott et al., 2010; Muoio et al., 2014). The coordination of neuronal and vascular function is essential to maintain cerebral activity because the nervous tissue has a very high metabolic rate that depends on the appropriate blood supply (Rolfe and Brown, 1997). Then, changes in neuronal activity must be paralleled by proportional and timely variations in blood flow to thereby match the metabolic demand. This is achieved by a communication mechanism that links neuronal and vascular function, which is known as neurovascular coupling (Iadecola, 2004; Hawkins and Davis, 2005; Leybaert, 2005; Koehler et al., 2006). In this context, an increment in synaptic activity rapidly leads to vasodilation of local parenchymal arterioles and, consequently, to an increase in blood-borne energy substrate that satisfies the enhanced metabolic demand (Anderson and Nedergaard, 2003; Iadecola, 2004; Leybaert, 2005).

**Figure 1:**
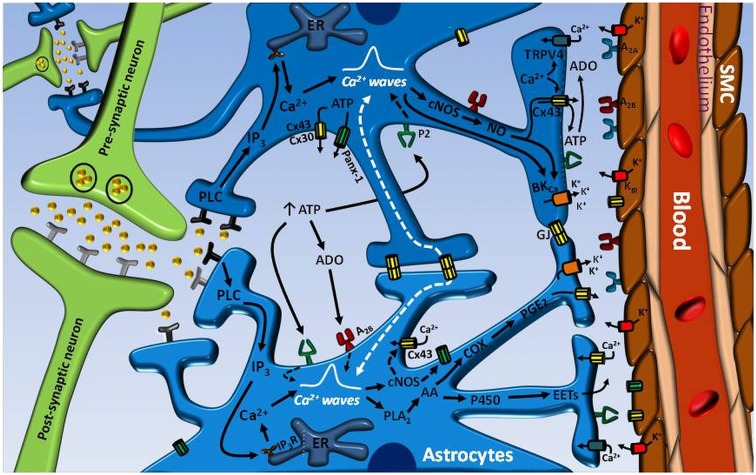
**Astrocyte-mediated signaling mechanisms involved in the control of neurovascular coupling**. Neurotransmitters released during an increase in neuronal activity can exit the synaptic cleft and activate receptors on astrocyte processes. The stimulation of astrocyte receptors initiates an inositol 1, 4, 5-triphosphate (IP_3_) receptor (IP_3_R)-mediated Ca^2+^ signal that is propagated through the astrocytic processes into the endfeet and activates the phospholipase A_2_ (PLA_2_)–arachidonic acid (AA) pathway and large conductance Ca^2+^-activated K^+^ channels (BK_Ca_). In turn, the activation PLA_2_–AA pathway leads to cytochrome P450 epoxygenase (P450)-mediated epoxyeicosatrienoic acids (EETs) production and cyclooxygenase (COX)-dependent prostaglandin E_2_ (PGE_2_) formation. Consequently, EETs and PGE_2_ release and BK_Ca_ channel opening evoke the vasodilation of parenchymal arterioles. The astrocyte-mediated vasodilator signal may be coordinated by the propagation of an inter-astrocyte Ca^2+^ signal via ATP release-mediated purinergic receptor (P_2_) stimulation or directly by gap junction communication (GJ). ATP may be released by either Cx30- or Cx43-based hemichannels or pannexin-1 (Panx-1)-formed channels. The hydrolysis of ATP to adenosine (ADO) by ecto-ATPases may also contribute to enhance and coordinate the Ca^2+^ signal through A_2B_ receptor activation on astrocytes. ADO formation from Cx43 hemichannel-driven ATP release at the endfeet may participate in the vasodilator response by the stimulation of A_2A_ receptors on vascular smooth muscle cells (SMC) of parenchymal arterioles. The astrocytic Ca^2+^ signal may also activates nitric oxide (NO) production by both Ca^2+^-dependent constitutive NO synthases (cNOS), eNOS and nNOS, which may play an important role in the regulation of neurovascular coupling by the activation of Cx43 hemichannels and BK_Ca_ channels. It is noteworthy that Cx43 hemichannel opening may contribute to the Ca^2+^ signal by providing a pathway for Ca^2+^ influx and, in addition, may participate in the astrocytic vasodilator mechanisms by allowing the efficient release of PGE_2_ and NO.

Although neurovascular coupling may be simply explained by the release of vasoactive signals from neurons, this signaling process is much more complex and astrocytes have emerged as central players in the communication of the changes in synaptic activity to local parenchymal arterioles (Anderson and Nedergaard, 2003; Koehler et al., 2006; Filosa and Iddings, 2013). Astrocytes are multifunctional cells that play a critical role in the maintenance of cerebral homeostasis and are in a strategic position to mediate and coordinate neurovascular coupling, since they are located between neurons and the microvasculature (Anderson and Nedergaard, 2003; Zonta et al., 2003a; Koehler et al., 2006; Filosa and Iddings, 2013; Figure 1). In this context, astrocytes project processes that surround neuronal synapses and express functional receptors for several neurotransmitters, which provides to these cells with a fine mechanism to sense neuronal activity (Porter and McCarthy, 1997; Zonta et al., 2003a; Koehler et al., 2006; Takano et al., 2006). Additionally, the astrocytic endfeet of the same astrocytes are found in an intimate, tight spatial organization with parenchymal vessels, where the endfeed reach to close proximity of the microvasculature and encase the vessel wall (Figure 1), which allows a fine and efficient communication between astrocytes and vascular cells (Anderson and Nedergaard, 2003; Iadecola, 2004; Koehler et al., 2006). This cellular spatial organization determines the basic signaling substrate for regulation of neurovascular coupling in which stimulation of receptors located at astrocyte processes by neurotransmitters released during an increase in neuronal activity initiates a Ca^2+^ signal that propagates through the astrocytic processes into the endfeet (Zonta et al., 2003a; Filosa et al., 2004; Koehler et al., 2006; Straub et al., 2006; Straub and Nelson, 2007). The generation of an astrocytic endfoot Ca^2+^ signal leads to the release of vasoactive factors that, in turn, evoke the arteriolar dilation required to produce an increase in local blood flow proportional to the increment in synaptic activity (Zonta et al., 2003a; Koehler et al., 2006; Takano et al., 2006; Straub and Nelson, 2007). Consistent with the astrocytic Ca^2+^ signaling-dependent control of arteriolar vasomotor tone, the increase in astrocytic endfoot cytosolic Ca^2+^ concentration ([Ca^2+^]_i_) precedes vasodilation of cerebral arterioles (Straub et al., 2006; Straub and Nelson, 2007; Filosa and Iddings, 2013).

Several Ca^2+^-dependent vasodilator mechanisms have been proposed to be activated at the astrocytic endfeet facing the arteriolar vessel wall. The most recognized vasodilator signals released from astrocytic endfeet are epoxyeicosatrienoic acid (EETs) and prostaglandin E_2_ (PGE_2_), which are synthesized by the cytochrome P450 epoxygenase and by a cyclooxygenase enzyme-dependent pathway, respectively (Figure 1), from the initial Ca^2+^-dependent arachidonic acid formation (Anderson and Nedergaard, 2003; Zonta et al., 2003a,b; Koehler et al., 2006; Straub and Nelson, 2007). Nevertheless, it must be noted that the release of arachidonic acid by astrocytes has been shown to lead to vasoconstriction through the production of 20-hydroxyeicosatetraenoic acid (20-HETE) in the arteriolar smooth muscle cells (Mulligan and MacVicar, 2004; Metea and Newman, 2006). This apparent controversy in the vascular response triggered by astrocytic Ca^2+^ signals (vasodilation vs. vasoconstriction) has been addressed in further studies. Girouard et al. (2010) show in mouse cortical brain slices that changes in the concentration of K^+^ ions in the space found between astrocytic endfoot and vessel wall may control the arteriolar vasomotor tone in a bimodal manner (i.e., generating vasodilation or vasoconstriction). Astrocytic endfeet express Ca^2+^-activated K^+^ channels of large conductance (BK_Ca_) and vascular smooth muscle cells of the parenchymal arterioles express inward rectifier K^+^ channels (K_ir_) (Price et al., 2002; Filosa et al., 2006; Girouard et al., 2010). Then, the increase in [Ca^2+^]_i_ generated in the endfeet during the neurovascular coupling triggers the opening of BK_Ca_, which leads to the release of K^+^ ion into the perivascular space, producing an increase in the local extracellular K^+^ concentration proportional to the magnitude of the Ca^2+^ signal that triggers the BK_Ca_ activation. Thereby, an increase in the perivascular K^+^ concentration smaller than 20 mM activates the K_ir_ channels located in the smooth muscle cell membrane facing the endfeet (Filosa et al., 2006; Girouard et al., 2010; Figure 1), leading to smooth muscle hyperpolarization, and consequently, vasodilation (Girouard et al., 2010). However, higher increases in extracellular K^+^ concentration (>20 mM) eliminates the electrochemical gradient of K^+^ and produces smooth muscle cell depolarization and vasoconstriction (Girouard et al., 2010). In addition, the direction of the vasomotor response initiated by the astrocytic endfoot Ca^2+^ signal has also been proposed to depend on the metabolic state of the tissue, which was evaluated by changing the oxygen tension in the superfusion solution of the experimental preparation. In this context, when hippocampal–neocortical slices were superfused with an artificial cerebrospinal fluid equilibrated with 95% O_2_, the response associated to the increase in astrocytic Ca^2+^ was vasoconstriction, but, in contrast, a vasodilation was activated in the presence of 20% O_2_ (Gordon et al., 2008; Attwell et al., 2010).

### Astrocytic Ca^2+^ signaling in neurovascular coupling

The activation of Ca^2+^ oscillations is a central signaling mechanism for astrocyte function and for transducing neuronal activity into vasodilation of parenchymal arterioles (Zonta et al., 2003a; Filosa et al., 2004; Straub et al., 2006; Straub and Nelson, 2007; Filosa and Iddings, 2013). The most relevant neuronal signal that triggers an increase in [Ca^2+^]_i_ in neurovascular coupling is the activation of metabotropic glutamate receptors located on astrocyte projections associated with glutamatergic synapses (Zonta et al., 2003a; Straub and Nelson, 2007; Filosa and Iddings, 2013). However, it should be noted that other neurotransmitters such as ACh, ATP and GABA or the release of neuropeptides such as somatostatine and vasoactive intestinal peptide from interneurons can also evoke the initiation of a Ca^2+^ signal in astrocytes (Stout et al., 2002; Li et al., 2003; Koehler et al., 2006; Straub et al., 2006). The synaptic activity-dependent activation of an astrocytic [Ca^2+^]_i_ is propagated as a Ca^2+^ wave along the perisynaptic astrocytic processes through the astrocyte to finally reach the perivascular endfeet (Zonta et al., 2003a; Filosa et al., 2004; Straub et al., 2006). The, apparently, most important and well-described mechanism involved in this Ca^2+^ signal is the activation of a phospholipase C (PLC)-dependent pathway, with the consequent generation of inositol 1, 4, 5-triphosphate (IP_3_) from membrane phospholipids, and then, the stimulation of Ca^2+^ release from the endoplasmic reticulum (ER) via IP_3_ receptors (IP_3_R; Parri and Crunelli, 2003; Straub et al., 2006; Straub and Nelson, 2007; Filosa and Iddings, 2013; Figure 1). However, PLC signaling also leads to diacylglycerol formation and protein kinase C (PKC) activation, which may also be involved in the modulation of Ca^2+^ oscillations as a negative feedback mechanism, since inhibition of PKC results in spontaneous [Ca^2+^]_i_ increases in slices of ventrobasal thalamus in resting conditions (Parri and Crunelli, 2003). Although Ca^2+^ release via ryanodine receptors was shown to contribute to the generation of astrocytic Ca^2+^ signals in cultured astrocytes (Golovina and Blaustein, 1997, 2000), the inhibition or activation of these receptors using ryanodine or caffeine, respectively, did not affect the characteristics of endfoot Ca^2+^ signals observed in native astrocytes in cortical slices (Straub et al., 2006).

Consistent with the participation of IP_3_R in the propagation of [Ca^2+^]_i_ increases into the astrocytic endfeet, spatially restricted photorelease of IP_3_ from caged IP_3_ within endfeet initiates a Ca^2+^ signal with similar characteristics to those observed in response to neuronal activation by electric field stimulation (Straub et al., 2006). Furthermore, the astrocytic endfoot [Ca^2+^]_i_ increase observed in both cases, IP_3_ uncaging and neuronal activation, was markedly blunted after the intracellular Ca^2+^ stores were depleted using cyclopiazonic acid (CPA), a blocker of the sarcoplasmic/ER Ca^2+^ ATPase (SERCA; Straub et al., 2006). Interestingly, Ca^2+^ signals initiated by photorelease of IP_3_ did not spread back to the cell body, but, as expected, this increase in [Ca^2+^]_i_ was associated with the initiation of a vasodilator response in the adjacent arteriole, suggesting that each endfoot works as a vasodilator unit in which the regenerative Ca^2+^ signaling machinery is spatially organized to direct the propagation of the Ca^2+^ oscillations in function of the activation of the Ca^2+^-dependent vasoactive signaling (Straub et al., 2006; Straub and Nelson, 2007). In addition, the intensity of the IP_3_R-generated Ca^2+^ signals associated to neuronal activation were heterogeneous throughout of the processes and endfeet, with spatially restricted regions of elevated [Ca^2+^]_i_ (Straub et al., 2006), which indicate that the generation of Ca^2+^ signals is a dynamic process with specialized points of amplification along the perivascular projections that may be associated with the activation of vasodilator signaling pathways during neurovascular coupling. Although these data confirm that IP_3_Rs play a central role in the generation and propagation of Ca^2+^ oscillations, it is important to note; however, that inhibition of IP_3_Rs with xestospongin only attenuates the Ca^2+^ signals generated in response to neuronal activation by electrical field stimulation in brain slices (Straub et al., 2006), and then, an additional mechanism may be involved in the response.

In addition to IP_3_R, astrocytic endfeet also express plasma membrane cation channels of the transient receptor potential vanilloid (TRPV) family (Nilius and Voets, 2005; Pedersen et al., 2005), specifically, the TRPV4 subtype (Benfenati et al., 2007) and Dunn et al. (2013) recently showed that stimulation of these channels with the agonist GSK1016790A increases the amplitude and frequency of spontaneous Ca^2+^ oscillations observed in cortical astrocytic endfeet of mouse coronal brain slices, which was associated with vasodilation of parenchymal arterioles. As expected, this response was absent in the presence of the TRPV4 antagonist HC-067047 or in TRPV4 knockout mice (Dunn et al., 2013). As IP_3_R in the ER membranes are activated by Ca^2+^, it is thought that propagation of Ca^2+^ waves is supported by a mechanism of Ca^2+^-induced Ca^2+^ release via IP_3_Rs in the ER membranes (Li et al., 2003; Parri and Crunelli, 2003; Straub et al., 2006), which seems to be enhanced through Ca^2+^ entry via TRPV4 channels (Dunn et al., 2013). Consistent with this notion, treatment with CPA reduced the amplitude, frequency and propagation distance of the GSK1016790A-induced endfoot Ca^2+^ oscillations observed in brain slices. The participation of IP_3_Rs in the effect of CPA was confirmed using xestospongin (Dunn et al., 2013). In addition, inhibition of TRPV4 channels with HC-067047 resulted in a reduction of the rise in endfoot [Ca^2+^]_i_ and the dilation of the associated parenchymal arteriole evoked by electrical field stimulation of brain slices from wild type animals, but not from TRPV4 knockout mice (Dunn et al., 2013). Interestingly, these results were confirmed in the intact animal through the evaluation of the cerebral hemodynamic response *in vivo* by measuring cerebral blood flow in the mouse somatosensory cortex using laser Doppler flowmetry in a cranial window. Although TRPV4 inhibition did not alter resting cerebral vascular function in this model, the evaluation of neurovascular coupling resulted in a reduction in the increase in cerebral blood flow observed in response to contralateral whisker stimulation (Dunn et al., 2013). These results indicate that TRPV4 channels are involved in the fine regulation of neurovascular coupling likely by interacting with the IP_3_R-mediated Ca^2+^ signals in the astrocyte endfeet.

### Connexins and pannexins in neurovascular coupling

An individual astrocyte connects multiple neuronal synapses with surrounding vessels and, conversely, an increase in neuronal activity is sensed by many astrocytes. Then, a single astrocyte must integrate the information of several neurons, but, in turn, the astrocyte-mediated neurovascular signaling must be coordinated between all astrocytes involved in the response to efficiently translate enhanced synaptic activity into higher blood flow to the whole brain region in which increased the metabolism (Araque et al., 1999; Haydon and Carmignoto, 2006). This tight and precise coordination of the astrocyte Ca^2+^ signaling generated by neuronal activation seems to be achieved, in great part, through connexin (Cxs)-mediated intercellular communication (Simard et al., 2003; Orellana et al., 2011).

Connexins belong to the protein family that forms the intercellular channels known as gap junctions, which communicate directly the cytoplasm of two neighboring cells, allowing intercellular transfer of current and solutes smaller than 1.4 nm of diameter (Perkins et al., 1998; Unger et al., 1999), such as ions and second messengers (e.g., Ca^2+^ and IP_3_) (Evans and Martin, 2002; Saez et al., 2003; Isakson et al., 2007). The association of six connexins makes up a hemichannel (i.e., half of gap junction channel) and head to head alignment of two hemichannels, each one provided by each adjacent cell, composes a gap junction channel (Saez et al., 2003). In addition to form gap junction channels, individual hemichannels are functional and provide a communication pathway between the intra and extracellular compartments, allowing influx of ions or release of paracrine/autocrine signals (Bruzzone et al., 2001; Stout et al., 2002; Goodenough and Paul, 2003; Cherian et al., 2005; Figueroa et al., 2013).

It has been described that astrocytes express several connexin isoforms, but Cx30 and Cx43 have been recognized as the most prominent connexins of these cells (Thompson and MacVicar, 2008; Ezan et al., 2012; Gaete et al., 2014). Although gap junctions provide a direct communication pathway for the propagation and coordination of Ca^2+^ signals between astrocytes (Simard et al., 2003; Orellana et al., 2011; Chandrasekhar and Bera, 2012), connexin hemichannels may also be involved in this process. Opening of Cx43-formed hemichannels is control by Ca^2+^ and these hemichannels are permeable to Ca^2+^ (De Bock et al., 2011, 2012; Chandrasekhar and Bera, 2012). Then, hemichannels may contribute to generate Ca^2+^ signals initiated by [Ca^2+^]_i_ increases as those observed in astrocytes in response to neuronal activation. In this context, Ca^2+^ oscillations activated by bradykinin in rat brain endothelial (RBE4) cells or Madin-Darby canine kidney (MDCK) cells were sensitive to short-time application (<30 min) of the connexin blocking peptides ^37,43^Gap27 (a mimetic peptide of the second extracellular loop of Cx37 and Cx43) or ^43^Gap26 (a mimetic peptide of the first extracellular loop of Cx43), respectively (De Bock et al., 2011, 2012). This rapid effect of connexin mimetic peptides is consistent with hemichannel inhibition, because gap junction function is only disrupted by longer periods of treatment. In addition, in MDCK cells, bradykinin-induced Ca^2+^ oscillations were also inhibited after reducing the extracellular Ca^2+^ concentration, siRNA silencing of Cx43 or altering the carboxy-terminal-dependent Ca^2+^-mediated regulation of Cx43 hemichannels by loading the cells with the peptide CT9 that correspond to the last 9 amino acids of the Cx43 carboxy-terminal (De Bock et al., 2012). As Ca^2+^ oscillations depend on IP_3_R activation and hemichannel opening by photolytic release of Ca^2+^ did not triggered Ca^2+^ oscillations (De Bock et al., 2012); these results show that Cx43-formed hemichannels may contribute to the generation of IP_3_R commanded Ca^2+^ signals, probably, by providing a pathway for Ca^2+^ stores refilling.

In addition, hemichannels formed by Cx30 and Cx43 have been described to be permeable to ATP (Stout et al., 2002; Kang et al., 2008; Sipos et al., 2009; Svenningsen et al., 2013) and ATP release has been shown to represent an important mechanism involved in the regenerative propagation of Ca^2+^ signals along the astrocyte processes and in the coordination of this signal between neighboring astrocytes (Stout et al., 2002; Orellana et al., 2011). Likewise Cx43 hemichannels, Cx30-based hemichannels may also be activated by Ca^2+^, and then, the increase in astrocytic [Ca^2+^]_i_ can lead to ATP release through Cx30 hemichannels or Cx43 hemichannels or both (Figure 1). The subsequent rise in extracellular ATP concentration can stimulate P2 purinergic receptors on either the same astrocyte from which it was released or on neighboring astrocytes (Simard et al., 2003; Suadicani et al., 2009; Orellana et al., 2011), which may contribute to enhance the Ca^2+^ wave propagation or to the intercellular coordination of the Ca^2+^ signaling, respectively. In addition of ATP release, the importance of connexins in neurovascular coupling is highlighted by the fact that Cx43 hemichannels were also found to mediate the release of PGE_2_ (Cherian et al., 2005; Figure 1).

It is noteworthy that astrocytes express pannexin-1 (Panx-1), a member of a protein family (Panx-1, Panx-2 and Panx-3) that forms channels with similar characteristics of connexin hemichannels (Panchin et al., 2000; Bruzzone et al., 2003). Panx-1-formed channels are not thought to contribute to gap junction-like communication, but they have been found to mediate ATP release in astrocytes (Iglesias et al., 2009; Orellana et al., 2011; Suadicani et al., 2012). Although there is an increasing body of evidence supporting the release of ATP via connexin hemichannels and pannexin channels, it is important to note that astrocytes may also release ATP by Ca^2+^-dependent exocytosis (Pryazhnikov and Khiroug, 2008). The relevance of ATP release in neurovascular coupling and the involvement of connexins, pannexins and exocytosis have not yet conclusively determined, but it is likely that, if these three mechanisms co-exist, they contribute to different phases of the response or are activated in distinct physiological conditions, which may provide fine regulation of ATP signaling in astrocytes.

Astrocytes and cerebral arterioles express adenosine receptors (Pilitsis and Kimelberg, 1998; Ngai et al., 2001) and ATP may rapidly be hydrolyzed to adenosine by extracellular ecto-ATPases (Xu and Pelligrino, 2007; Pelligrino et al., 2011; Vetri et al., 2011), which, in astrocytes, have been described to be located close to hemichannels (Joseph et al., 2003; Fields and Burnstock, 2006). Then, the ATP hydrolysis to adenosine may also contribute to the propagation and coordination of astrocyte-mediated Ca^2+^ signals and directly to the dilation of parenchymal arterioles in response to neuronal activation (Figure 1). Interestingly, activation of A_2B_ receptors has been reported to elicit an increase in [Ca^2+^]_i_ (Pilitsis and Kimelberg, 1998) and potentiate the ATP-induced Ca^2+^ response in astrocytes (Jiménez et al., 1999; Alloisio et al., 2004). Consistent with the participation of these receptors in neurovascular coupling, A_2B_ antagonists inhibit the increase in cerebral blood flow observed in response to whisker stimulation (Shi et al., 2008). In addition, adenosine derived from ATP released via connexin hemichannels located at astrocyte endfeet (Simard et al., 2003) may evoke arteriolar dilation by direct stimulation of vascular smooth muscle A_2A_ or A_2B_ receptors (Ngai et al., 2001), which is coherent with the inhibition by A_2A_ antagonists of the pial arteriolar dilation observed during sciatic nerve stimulation (Meno et al., 2001).

### Nitric oxide (NO) in neurovascular coupling

Nitric oxide (NO) is a widely distributed, pleiotropic signaling molecule synthesized by the enzyme NO synthase (NOS) from the amino acid L-arginine (Moncada et al., 1991). Three isoforms of NOS have been described: endothelial NOS (eNOS), neuronal NOS (nNOS) and inducible NOS (iNOS; Moncada et al., 1991; Alderton et al., 2001). eNOS and nNOS are expressed constitutively primarily, but not exclusively, in endothelial cells and neurons, respectively, and the activation of these isoforms depends on an increase in [Ca^2+^]_i_ (Alderton et al., 2001). In contrast, the expression of iNOS is typically assumed to be induced by cytokines and others agents during the immune response and its activity does not depend on an increment in [Ca^2+^]_i_ (Pautz et al., 2010). NO is a potent vasodilator (Moncada et al., 1991), which led to the proposal that neurovascular coupling is directly mediated by the Ca^2+^-dependent NO production associated to the activation of cortical neurons. In fact, inhibition of NO production with N^G^-nitro-L-arginine (L-NA, a general NOS inhibitor), deletion of nNOS and specific nNOS inhibition with 7-nitroindazole have been reported to attenuate the increase in sensory cortex cerebral blood flow observed in response to vibratory hindpaw stimulation in mouse (Kitaura et al., 2007) or transcallosal electrical stimulation *in vivo* in rat (Brožíčková and Otáhal, 2013). Although these data support the participation of nNOS in neurovascular coupling, they are not in disagreement with the critical role played by astrocytes in this response, because NO-synthesizing enzymes are not present in excitatory neurons of many brain regions (Iwase et al., 1998; Karagiannis et al., 2009; Tricoire et al., 2010) and astrocytes have been shown to express eNOS and nNOS (Gabbott and Bacon, 1996; Doyle and Slater, 1997; Shin, 2001; Lin et al., 2007). Additionally, astrocytes may also express low levels of iNOS, which has also been related with normal astrocyte function (Buskila et al., 2007). NO production by astrocytes has been proposed to participate in the regulation of neuronal activity (Buskila et al., 2007), astrocytic spontaneous Ca^2+^ transients (Schipke et al., 2008) and the astrocytic release of glutamate and ATP (Bal-Price et al., 2002; Ida et al., 2008).

It is well-known that the effects of NO are mediated by the activation of the soluble guanylate cyclase and the cGMP/PKG pathway, which has been considered as the “classical” mechanism of NO signaling (Moncada et al., 1991). Nevertheless, beside activation of soluble guanylate cyclase, S-nitrosylation (also termed as S-nitrosation) has emerged as an important “non-classical” mechanism of NO signaling (Ahern et al., 2002; Martínez-Ruiz et al., 2013). It is important to note that, in contrast to the activation of the cGMP/PKG pathway, the S-nitrosylation signaling mechanism is preferentially observed close to the NO source, where NO concentration is higher (Martínez-Ruiz et al., 2013). S-nitrosylation comprises NO-mediated oxidation of cysteine residues to form a nitrosothiol, a post-translational modification that has been recognized to modulate the activity of several signaling proteins (Martínez-Ruiz et al., 2013). As a physiological signaling process, S-nitrosylation is transient and the nitroso group can be removed (i.e., denitrosylation) after the stimulation fades out (Martínez-Ruiz et al., 2013; Sengupta and Holmgren, 2013). Interestingly, connexin function is regulated by S-nitrosylation (Retamal et al., 2006). In astrocytes, Cx43 was found to be S-nitrosylated in response to metabolic inhibition, which was tightly related to opening of hemichannels formed by this connexin isoform (Retamal et al., 2006). This finding is coherent with the recent demonstration that NO opens hemichannels expressed in cultured astrocytes and in HeLa cells transfected with Cx37, Cx40 or Cx43 (Figueroa et al., 2013), which shows that, in addition of Cx43 hemichannels, NO also induces the activation of Cx37- and Cx40-based hemichannels. Interestingly, this work also demonstrated that NO crosses the plasma membrane preferentially through connexin hemichannels (Figueroa et al., 2013), at least, through those formed by Cx37, Cx40 or Cx43. On the other hand, the effect of NO on Panx-1-formed channels is controversial, since NO has been found to activate or inhibit these channels and in both cases S-nitrosylation was proposed to be involved (Zhang et al., 2008; Lohman et al., 2012).

The potential relevance of NO-induced connexin hemichannel activation in neurovascular coupling is highlighted by the contribution of NO to the ATP-elicited Ca^2+^ signal in astrocytes that described Li and collaborators (Li et al., 2003). These authors found that the release of Ca^2+^ from the intracellular stores initiated by ATP leads to the activation of a NO-dependent pathway of Ca^2+^ influx that plays an important role in the increase in [Ca^2+^]_i_ and the subsequent Ca^2+^ store refilling observed in this response. The NO-induced Ca^2+^ influx did not depend on the activation of cGMP production (Li et al., 2003), suggesting the involvement of S-nitrosylation. Interestingly, the Ca^2+^ influx activated by NO was sensitive to Cd^2+^ and 2-aminoethoxydiphenyl borate (2-APB; Li et al., 2003). Although Cd^2+^ is thought to be a nonselective Ca^2+^ channel blocker and 2-APB is recognized as an IP_3_R antagonist, both blockers have been shown to inhibit connexin hemichannels (Tao and Harris, 2007; Tang et al., 2009). Then, these results suggest that NO-dependent connexin hemichannel activation by S-nitrosylation may be involved, not only in ATP release, but also in the Ca^2+^ signaling evoked by ATP in astrocytes, and consequently, in the Ca^2+^ wave propagation observed in the neurovascular coupling (Figure 1), which is consistent with the recent report indicating that inhibition or deletion of eNOS blunted the astrocyte-mediated neurovascular coupling-dependent vasodilation (Stobart et al., 2013). Furthermore, as connexin hemichannels mediate the intercellular transfer of NO (Figueroa et al., 2013) and Cx43 is preferentially expressed in astrocytic endfeet (Simard et al., 2003), Cx43-formed hemichannels may contribute to the neuronal activation-induced vasodilation by directing the NO signaling toward parechymal arterioles (Figure 1). In addition of connexins, NO signaling has also been shown to be involved in the control of TRPV4 and BK channel function. NO regulates negatively TRPV4 channels by S-nitrosylation (Lee et al., 2011) and induces the opening of BK directly by S-nitrosylation or through the cGMP/PKG pathway (Bolotina et al., 1994; Tanaka et al., 2000), which suggests that NO may regulate the astrocytic Ca^2+^ signaling at different levels and contribute to the BK-mediated vasodilation (Figure 1).

Although opening and regulation of connexin hemichannels is not yet clear in the context of astrocyte function in normal physiological conditions, these data suggest that Ca^2+^-mediated activation of NO production may be involved in the regulation of the astrocytic Ca^2+^ signal triggered in neurovascular coupling through activation of a Ca^2+^ influx or ATP release via Cx43-formed hemichannels. However, the involvement of connexin hemichannels or Panx-1 channels in the NO-dependent regulation of the neuronal activation-initiated Ca^2+^ and ATP signaling in astrocytes remains to be determined.

## Concluding remarks

Neurovascular coupling is a complex signaling mechanism that depends on functional and coordinated interactions of astrocyte with neurons and vascular cells. Changes in neuronal activity are transduced into vasomotor responses through astrocytic Ca^2+^ signals, which are activated by the neurotransmitters released at the synapsis, principally glutamate. The Ca^2+^ signal is propagated through the astrocytic processes to the endfeet by an IP_3_R-dependent Ca^2+^-induced Ca^2+^ release mechanism and by autocrine ATP signaling via P2 purinergic receptors or A_2B_ adenosine receptors (after ATP hydrolysis by ecto-ATPases). ATP may be released through hemichannels formed by Cx30 or Cx43 and/or channels formed by Panx-1 and, in addition, activation of these channels provides a direct pathway for Ca^2+^ influx that may be involved in the regulation of the IP_3_R-initiated astrocytic Ca^2+^ signal. However, although connexins and Panx-1 are likely to play a central role in the astrocyte-mediated neurovascular coupling, NO seems to control and orchestrate the development of the Ca^2+^ response, since NO production is activated by the initial IP_3_R-mediated Ca^2+^ release and NO is involved in the generation, propagation and regulation of the Ca^2+^ signaling. This is because the increase in NO concentration leads to ATP release and activates a Ca^2+^ influx pathway that contributes to the astrocytic Ca^2+^ signal observed in response to both ATP or metabotropic glutamate receptor stimulation. The NO-evoked Ca^2+^ influx seems to be also involved in the regulation of the Ca^2+^ signaling by contributing to refill the IP_3_R-associated intracellular Ca^2+^ store. Although the activation of Cx43 hemichannels by S-nitrosylation may provide the pathway for the NO-dependent ATP release and Ca^2+^ influx, the participation of connexin- or Panx-1 formed channels in the NO-dependent Ca^2+^ signals must be confirmed in future investigations.

The propagation of the neuronal-activated Ca^2+^ wave into the astrocyte endfeet is supported and regulated by specialized signaling mechanisms of these subcellular domains. Astrocyte endfeet express Cx43 hemichannels and TRPV4 channels and although the generation of the Ca^2+^ signal in the endfeet is governed by IP_3_Rs, Ca^2+^-dependent activation of Cx43 hemichannels and TRPV4 channels may contribute to enhance the Ca^2+^ signal at specialized microdomains associated with the activation of vasodilator mechanisms. Interestingly, diffusion or production of NO in the endfeet may be involved in the control of the Ca^2+^ signal by inducing the opening of Cx43 hemichannels and the inhibition of TRPV4 channels. Furthermore, the NO-mediated Cx43 hemichannel activation may also play an important role in the astrocyte endfoot-elicited vasodilation by providing the pathway for the release of NO and PGE_2_ into the perivascular space. In addition of Cx43 hemichannels, NO may also induce the activation of BK channels at the astrocytic enfeet, which highlights the relevance of the interaction between NO and Ca^2+^ in the regulation of the astrocyte-dependent vasodilator signals activated during neurovascular coupling. The specific contribution of eNOS and nNOS to the astrocyte-conducted Ca^2+^-mediated vasodilator signaling may be determined by the subcellular location and spatial organization of these NOS isoforms in relation to other signaling proteins involved in the regulation of neurovascular coupling. Then, the study of the subcellular distribution of eNOS and nNOS in astrocytes and the possible association of these NO-synthesizing enzymes with connexins, Panx-1, TRPV4 channels and BK channels may be an interesting and fruitful area of investigation that may help to understand the complex and dynamic regulation of neurovascular coupling.

## Conflict of interest statement

The authors declare that the research was conducted in the absence of any commercial or financial relationships that could be construed as a potential conflict of interest.
